# A quantitative analysis of carbon-ion beam-induced reactive oxygen species and redox reactions

**DOI:** 10.3164/jcbn.18-34

**Published:** 2019-03-29

**Authors:** Ken-ichiro Matsumoto, Minako Nyui, Megumi Ueno, Yukihiro Ogawa, Ikuo Nakanishi

**Affiliations:** 1 Quantitative RedOx Sensing Team, Department of Basic Medical Sciences for Radiation Damages, National Institute of Radiological Sciences, National Institutes for Quantum and Radiological Science and Technology, 4-9-1 Anagawa, Inage-ku, Chiba 263-8555, Japan

**Keywords:** carbon-ion beam, hydroxyl radical, hydroperoxyl radical, hydrogen peroxide, electron paramagnetic resonance

## Abstract

The amounts of reactive oxygen species generated in aqueous samples by irradiation with X-ray or clinical carbon-ion beams were quantified. Hydroxyl radical (^•^OH), hydrogen peroxide (H_2_O_2_), and the total amount of oxidation reactions, which occurred mainly because of ^•^OH and/or hydroperoxy radicals (HO_2_^•^), were measured by electron paramagnetic resonance-based methods. ^•^OH generation was expected to be localized on the track/range of the carbon-ion beam/X-ray, and mM and M levels of ^•^OH generation were observed. Total ^•^OH generation levels were identical at the same dose irrespective of whether X-ray or carbon-ion beam irradiation was used, and were around 0.28–0.35 µmol/L/Gy. However, sparse ^•^OH generation levels decreased with increasing linear energy transfer, and were 0.17, 0.15, and 0.09 µmol/L/Gy for X-ray, 20 keV/µm carbon-ion beam, and >100 keV/µm carbon-ion beam sources, respectively. H_2_O_2_ generation was estimated as 0.26, 0.20, and 0.17 µmol/L/Gy, for X-ray, 20 keV/µm carbon-ion beam, and >100 keV/µm carbon-ion beam sources, respectively, whereas the ratios of H_2_O_2_ generation per oxygen consumption were 0.63, 0.51, and 3.40, respectively. The amounts of total oxidation reactions were 2.74, 1.17, and 0.66 µmol/L/Gy, respectively. The generation of reactive oxygen species was not uniform at the molecular level.

## Introduction

There are two mechanisms by which ionizing radiation generates biological effects—direct actions and indirect actions.^(1–3)^ Indirect actions are mediated by reactive oxygen species (ROS) or other free radical species generated by the radiolysis of water.^(4–6)^ Therefore, indirect actions are a chemical trigger of biological reactions, whereas direct actions are a physiological and stochastic trigger of the reactions. It is well known that ROS and/or free radicals can oxidize bio-functional molecules and induce oxidative molecular injury. As a consequence, abnormalities can occur in cellular systems.

Low linear energy transfer (LET) ionizing radiation has relatively large oxygen effects because of indirect actions mediated by ROS.^(7)^ However, high LET beams give relatively low oxygen effects compared with low LET radiation,^(8,9)^ and ROS generation with high LET beams has not been considered as a severe problem until recently. When clinical doses become higher in low-fractionated high LET protocols, however, the generation of ROS with high LET beams can become non-negligible. Therefore, the amount and distribution of ROS generated by clinical carbon-ion beams will be essential information for developing advanced carbon-ion beam therapy protocols in the future. Important ROS forms in radio-biological effects include hydroxyl radicals (^•^OH), hydrogen peroxide (H_2_O_2_), and superoxide (O_2_^•−^).

^•^OH, which is one of initial species generated by radiolysis of water, can be measured by electron paramagnetic resonance (EPR) spin trapping methods.^(10,11)^ For quantification, a sufficient amount of spin trapping agent must be added to the reaction system. When the concentration of spin-trapping agent was increased step-by-step and beyond the concentration of the target radical generation, the production of radical-spin-adducts should be saturated. In a previous report,^(12)^ several concentrations of spin-trapping agent solutions were prepared and irradiated with an identical dose of X-ray or carbon-ion beam irradiation. The result showed that relatively sparse (≈︀3.3 mol/L) and extremely dense (1.7 mol/L or more) localized ^•^OH generation levels were observed. The sparse ^•^OH generation decreased with increasing LET, but both the sparse and dense ^•^OH generation levels were independent of the dose, dose ratio, and energy of irradiation.^(13)^

H_2_O_2_ is a less reactive species compared with ^•^OH. Such low reactivity allows H_2_O_2_, which is a membrane permeable molecule, to travel a relatively long distance. In addition, H_2_O_2_ can be easily reduced by Fe^2+^ and/or Cu^+^ to give ^•^OH, the strongest oxidant among ROS. X-ray-induced H_2_O_2_ generation was estimated as 0.2 µmol/L/Gy in a previous report.^(14)^ The reduction of 4-hydroxy-2,2,6,6-tetramethylpiperidine-*N*-oxyl (TEMPOL), which can be observed at a relatively higher dose, was depressed by catalase. In other words, TEMPOL reduction can be replaced by H_2_O_2_ generation in the sample.^(14)^

Superoxide (O_2_^•−^) equilibrates with hydroperoxyl radicals (HO_2_^•^) in an aqueous environment. In other words, O_2_^•−^ and HO_2_^•^ coexist at any moment in an aqueous environment. HO_2_^•^ is a quite strong oxidant, and can oxidize biological molecules, even though O_2_^•−^ is essentially a reductant. A nitroxyl radical, such as TEMPOL, can be one-electron oxidized to give an oxoammonium cation by an oxidative ROS such as ^•^OH or HO_2_^•^. If in coexistence with a reduced form glutathione (GSH), the oxoammonium cation can then be reduced to hydroxylamine by receiving a hydrogen atom from glutathione, which is the main case in an *in vivo* situation,^(15–17)^ or reduced to make a stable compound with glutathione, which is the main case in *in vitro* conditions.^(18,19)^ The latter reaction is irreversible. Therefore, the amount of total oxidation can be measured using the amount of GSH-dependent reduction of TEMPOL in *in vitro* conditions. The amount of total oxidation reactions was estimated as 3 µmol/L/Gy for X-ray in a previous study.^(20)^

In this paper, a breakdown of the amounts of ROS, i.e., ^•^OH generation, H_2_O_2_ generation, and total oxidation reactions, in aqueous samples induced by irradiation with a clinical carbon-ion beam were compared. The distributions of ^•^OH generation, H_2_O_2_ generation, and total oxidation reactions induced by carbon-ion beam irradiation in a solid sample were also estimated and compared.

## Materials and Methods

### Chemicals

5,5-Dimethyl-1-pyrroline-*N*-oxide (DMPO) was purchased from Dojindo Laboratories, Ltd. (Kumamoto, Japan). TEMPOL (4-hydroxy-2,2,6,6-tetramethylpiperidine-*N*-oxyl) was purchased from Sigma-Aldrich Co. (St. Louis, MO). GSH and 30% H_2_O_2_ solution was purchased from Wako Chemical Co. (Tokyo, Japan). Other chemicals were of analytical grade. As the basic solvent for the reaction mixtures, 100 mmol/L phosphate buffer containing 0.05 mmol/L diethylenetriaminepentaacetic acid (DTPA) was prepared at pH 7.0 and used for all experiments. Deionized water (Milli-Q system, Merck Millipore, Billerica, MA) was used for preparing the 100 mM phosphate buffer.

### Measurement of ^•^OH

^•^OH was measured using an EPR spin-trapping method with DMPO as the spin-trapping agent. The theory and detailed procedures were described previously.^(12)^ A series of reaction mixtures containing several concentrations (0.5, 1.6, 3.3, 7.7, 13.3, 26.0, 61.6, 208, 600, and 1,685 mmol/L) of DMPO were prepared. For X-ray irradiation, a 150 µl aliquot of each concentration of reaction mixture was transferred to a polyethylene microtube, and kept on ice until irradiation. For carbon-ion beam irradiation, a 350 µl aliquot of the reaction mixture was transferred into a thin, flat, polyethylene bag, and kept on ice until irradiation. Then, 32 Gy of X-ray or a specified LET carbon-ion beam was irradiated onto the sample reaction mixtures using the conditions described below. For X-ray-irradiated samples, EPR measurements were started approximately 3 min after irradiation, and repeated every 1 min for 10 min. The reduction of DMPO-OH during the irradiation was corrected by an iterative calculation using the decay rate of DMPO-OH, as described previously.^(21)^ For the carbon-ion beam-irradiated samples, a single EPR measurement was performed for each sample 20~60 min after irradiation. Decay correction for carbon-ion beam irradiated samples were done as described previously.^(12)^ The EPR conditions are described below. 2.3 and 3.2 mol/L DMPO solution was also irradiated by X-ray and measured as same as above.

### Measurement of H_2_O_2_

H_2_O_2_ generations in irradiated samples were estimated by several methods below and compared.

1) Radiation-induced TEMPOL reduction in water, which was H_2_O_2_ related reaction, was ascribed to an index of H_2_O_2_ generation.^(14)^ An aqueous solution of 0.1 mmol/L TEMPOL was prepared. For X-ray irradiation, a 150 µl aliquot of each concentration of reaction mixture was transferred into a polyethylene microtube. For carbon-ion beam irradiation, a 350 µl aliquot of the reaction mixture was transferred into a thin, flat polyethylene bag. The sample reaction mixtures were kept at room temperature until irradiation. Then, 128 Gy of X-rays or a specified LET carbon-ion beam was used to irradiate the sample reaction mixtures in the conditions described below. The irradiated samples were measured using an X-band EPR in the conditions described below as soon as possible.

2) The ^•^OH synthesized from H_2_O_2_ under the presence of Fe^2+^ was spin-trapped with DMPO, and the ^•^OH adduct of DMPO (DMPO-OH) was then measured as an index of H_2_O_2_ using an X-band EPR.^(22,23)^ Irradiated water sample (160 µl) was placed in a microtube, and then 500 mmol/L DMPO (20 µl) was added to the microtube. When 1.0 mmol/L FeSO_4_ (20 µl) was added, the sample was immediately mixed, transferred to a PTFE tubing, and measured with an X-band EPR spectrometer (RE-100, JEOL, Tokyo) within a minute of mixing. EPR conditions were described as below. The H_2_O_2_ concentration was calculated based on the standard curve obtained previously using a concentration series of H_2_O_2_ solutions, which were made by diluting 30% H_2_O_2_ solution with ultrapure water at several concentrations (0–980 µmol/L).

3) H_2_O_2_ concentration in the irradiated water samples were measured using the spectrophotometric method.^(24)^ The method is based on measurement of a red quinoid dye formed by a reaction of 4-aminoantipyrine and phenol and H_2_O_2_ under coexisting peroxidase. This quinoid dye can be detected by absorbance at 505 nm using a spectrophotometer.

### TEMPOL decay in high concentration H_2_O_2_

X-band EPR signal intensities of reaction mixtures containing 0.1 mmol/L TEMPOL and several concentrations of H_2_O_2_ were measured before and after UV irradiation at room temperature. Decay rates of X-band EPR signal intensities of reaction mixtures containing 0.1 mmol/L TEMPOL and several concentrations of H_2_O_2_ were measured after 1 mmol/L K_3_Fe(CN)_6_ was added at room temperature.

### Measurement of total oxidation

Total oxidation levels were estimated by GSH-dependent TEMPOL reduction.^(20)^ An aqueous solution containing 0.1 mmol/L TEMPOL and 1 mmol/L GSH was prepared. For X-ray irradiation, a 150 µl aliquot of each concentration of reaction mixture was transferred into a polyethylene microtube. For carbon-ion beam irradiation, a 350 µl aliquot of reaction mixture was transferred into a thin, flat polyethylene bag. The sample reaction mixtures were kept on ice until irradiation. Either 16 Gy of X-rays or a specified LET carbon-ion beam was used to irradiate the sample reaction mixtures in the conditions described below. The irradiated samples were then kept on ice until EPR measurement. The irradiated samples were measured as soon as possible using an X-band EPR in the conditions described below.

### X-Ray Irradiation

X-ray irradiation was performed using a PANTAK 320S (Shimadzu, Kyoto, Japan) at room temperature. The effective energy of the X-ray was 80 keV with conditions set at: X-ray tube voltage, 200 kV; X-ray tube current, 20 mA; and thickness and materials of the pre-filter; 0.5 mm copper and 0.5 mm aluminum. The dose rate of X-ray irradiation was 3.2–3.3 Gy/min when the distance between the X-ray tube and the sample was 30 cm.

### Carbon-ion beam irradiation

The polyethylene bag containing the reaction mixture was attached to a flat acrylic sample holder and irradiated with a 290 MeV/nucleon carbon-ion beam using the Heavy-Ion Medical Accelerator in Chiba (HIMAC, National Institute of Radiological Sciences, Chiba, Japan) at room temperature. Irradiation experiments were performed using several LET conditions (13–190 keV/µm). The calculated LET at the surface of the sample was based on the thickness of the binary filter and the polyethylene wall. The dose was planned at the surface of the sample, and the dose rates varied depending on the LET.

### X-band EPR measurement

A 100 µl aliquot of irradiated reaction mixture was drawn into PTFE tubing (i.d. 0.32 ± 0.001 inches, wall 0.002 ± 0.0005 inches; ZEUS, Orangeburg, SC). The PTFE tubing was set in a TE-mode EPR cavity using a quartz sample tube, and then measured using an X-band EPR spectrometer (JEOL, Tokyo, Japan). EPR conditions were as follows: microwave frequency, 9.4 GHz; microwave power, 2.00 mW; main magnetic field, 337.9 mT; field modulation frequency, 100 kHz; field modulation amplitude, 0.063 mT; time constant, 0.3 s; and magnetic field sweep rate, 2.5 mT/min. For the measurement of DMPO-OH, only the second peak from the lower field was measured. For the measurement of TEMPOL, only the center peak was measured.

## Results and Discussion

**Fig. 1:**
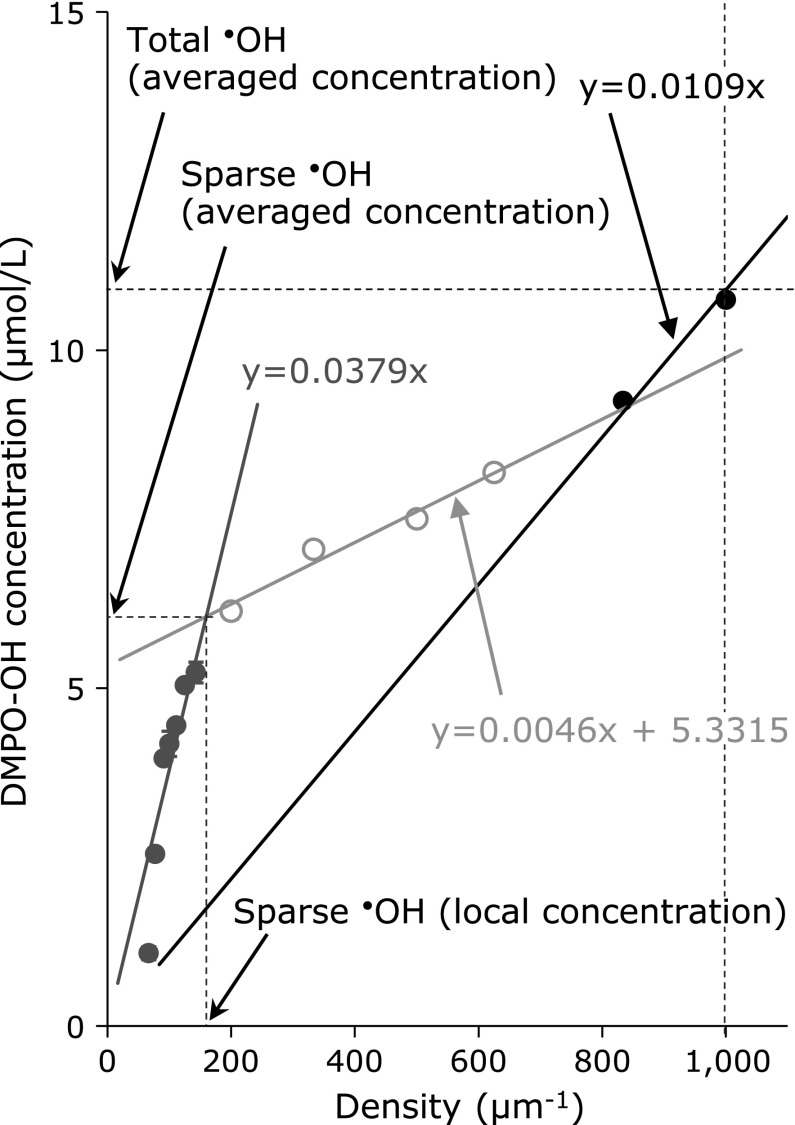
A typical profile of a plot of DMPO-OH concentration vs DMPO density obtained for a series of DMPO solutions after X-ray irradiation.

**Fig. 2:**
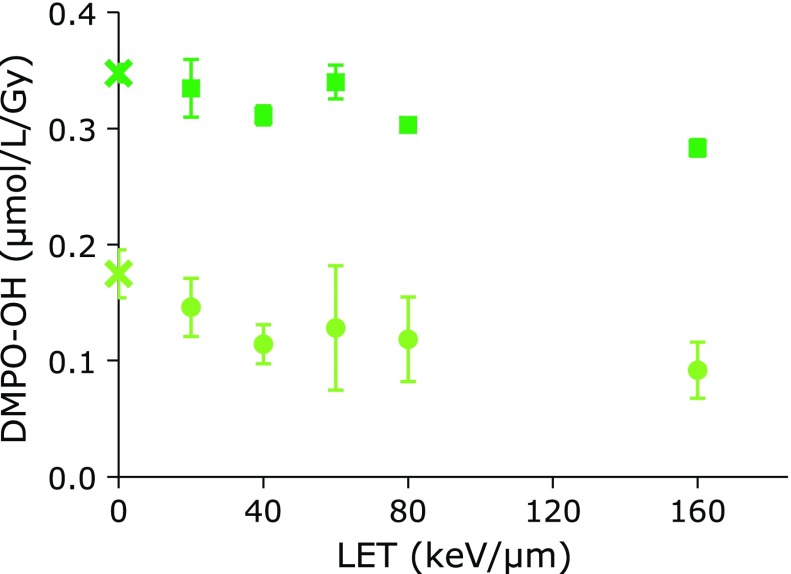
LET dependences of dense and sparse ^•^OH generation obtained for carbon-ion beams. Squares indicate total ^•^OH generations, which were obtained as the calculated Y-values at the 1,000 µm^−1^ of DMPO density on the least square line for the 3rd phase shown in Fig. 1. Circles indicate sparse ^•^OH generations which were obtained as the Y-values at the close point of least square fit for 1st and 2nd phases shown in Fig. 1. The marks and bars indicate averages and SD of 2 separate experiments. X on the Y-axis indicates values obtained for X-ray.

Figure 1 shows a plotted profile of DMPO-OH concentration vs DMPO density obtained for a series of DMPO solutions after X-ray irradiation. The plot shows a typical three phase profile. The first phase was a linear increasing phase through the origin (gray circles), and the second phase was a plateau-like phase (open circles). The third phase was another linear increasing phase through the origin (black circles), which indicated the extremely dense ^•^OH generation, at an expected rate of more than 1,000 µm^−1^ (>1,700 mmol/L). The intersection point of the least square lines obtained for the first and second phases was calculated by estimating the density (local concentration) and the amount (averaged mole number per volume) of sparse ^•^OH generation. For a case of X-ray (Fig. 1), the density of sparse ^•^OH generation was 160 µm^−1^, which corresponded to a concentration of 6.8 mM, and amount of sparse ^•^OH generation was 6.1 µmol/L (0.19 µmol/L/Gy). The DMPO-OH concentration at a DMPO density of 1,000 µm^−1^ was replaced with total ^•^OH generation, which was 11.3 µmol/L (0.35 µmol/L/Gy) in this case. The results of carbon-ion beam irradiation are summarized in Table 1 and Fig. 2.

Figure 2 shows the LET dependences of dense and sparse ^•^OH generation, respectively. The amounts of total ^•^OH generated were the similar among X-ray and all LETs of carbon-ion beam irradiation. The average value of total ^•^OH generation for carbon-ion beam irradiation experiments was 0.31 ± 0.023 µmol/L/Gy. However, the amounts for sparse ^•^OH generation seemed to decrease with increasing LET. The sparse ^•^OH generation at LET = 160 keV/µm was almost half of that at LET = 20 keV/µm. As shown in Table 1, the density of sparse ^•^OH generation was similar among X-ray and all LETs of carbon-ion beam irradiation. The average value of sparse ^•^OH density for carbon-ion beam irradiation experiments was 147 ± 8 µm^−1^, which corresponded to 5.6 ± 0.99 mmol/L.

The quantification ability of EPR spectroscopy is originally low. The techniques for quantification of free radicals using internal and external standard sample is still developing but not 100% perfect. The values reported for DMPO-OH concentrations in the previous paper^(12)^ were in the same order but somewhat different from the values estimated in this paper. The trend of values observed, i.e., three-phase patterns of the plot of DMPO-OH concentration vs DMPO density, and the LET dependent behaviors were the same as the previous paper. It could be 0.29 µmol/L/Gy, when an ^•^OH generation was calculated from the g-value, 2.82, reported for photon.^(25)^ The total ^•^OH generation obtained in this paper was much closer to the calculated value.

**Fig. 3:**
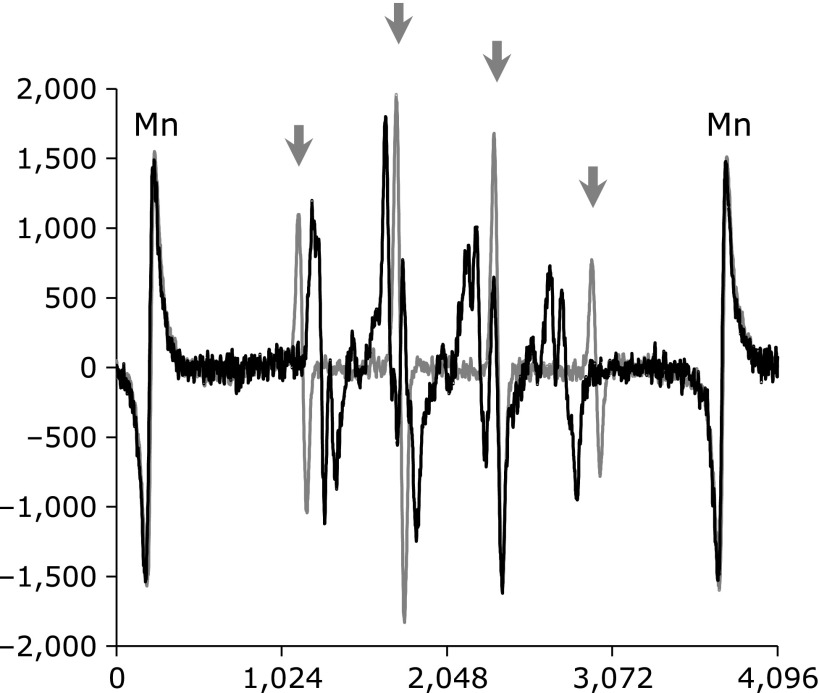
EPR spectrum obtained after X-ray irradiation to the 99% DMPO (black line). The gray line indicates typical EPR spectra of DMPO-OH. Both spectra include 3rd and 4th EPR lines of Mn-maker on the edge. Arrows indicate the position of 1:2:2:1 quartet signal of DMPO-OH.

The solid black line in Fig. 3 shows an EPR spectrum caused in 99.0% DMPO (undiluted commercial liquid) irradiated by 32 Gy X-ray. This spectrum, which is named DMPO-irradiated for convenience for here, is totally different from an EPR spectrum of DMPO-OH (gray line). The X-ray induced EPR spectrum caused in 1.7 mol/L DMPO was looked like a DMPO-OH. However, the overlapping spectrum of DMPO-OH and DMPO-irradiated when 2.3 or 3.2 mol/L DMPO was irradiated by X-ray, and then the ratio of DMPO-irradiated was increased in the 3.2 mol/L DMPO compared to the 2.3 mol/L DMPO. This result suggests that generation of DMPO-OH requires water.

It is known that DMPO-OH can be formed by decomposition of the superoxide adduct of DMPO (DMPO-OOH). The radiation induced O_2_^•−^ is probably generated by a reaction of hydrogen radical (^•^H) and molecular oxygen (O_2_) as indicated by Eq. 1 and 2.

^•^H + O_2_ → HO_2_^•^    [1]

HO_2_^•^ ⇄ O_2_^•−^ + H^+^    [2]

Since the spin trapping agents can trap hydrogen radical, the O_2_^•−^ generation was almost eliminated in this method at all.

A sequence of radiation induced ionizations probably be going on a line, which may be not always straight, somewhat zigzag, and having some branches. Only a DMPO happen to be close to the ^•^OH generated can be reacted to trap the ^•^OH localized on a branch-like breadth, but most of DMPO in a 3D volume are not able to reach the ^•^OH before the ^•^OH was lapsed. Therefore, the number of detectors on a linear distance, which was defined as “density” in our previous paper,^(12)^ should be directly indexed to estimate number of ^•^OH generated with such sequential ionizations, while the number of DMPO in a 3D volume, i.e., concentration, is insensitive to estimate such a localized ^•^OH generation.

The exact sample volume occupied the detectable volume in the EPR cavity resonator was unknown. The same volume sample with known spin concentration was used as the standard to calculate the DMPO-OH concentration generated in the irradiate sample. An exactly prepared 2.0 mmol/L TEMPOL water solution was used as a standard sample in this paper. The spin-trapped ^•^OH, i.e., DMPO-OH, were only estimated by concentration, which means number of molecules in a 3D volume of the sample. However, it is not a matter to estimate number of DMPO-OH even localized or equally-distributed in the sample volume.

**Fig. 4:**
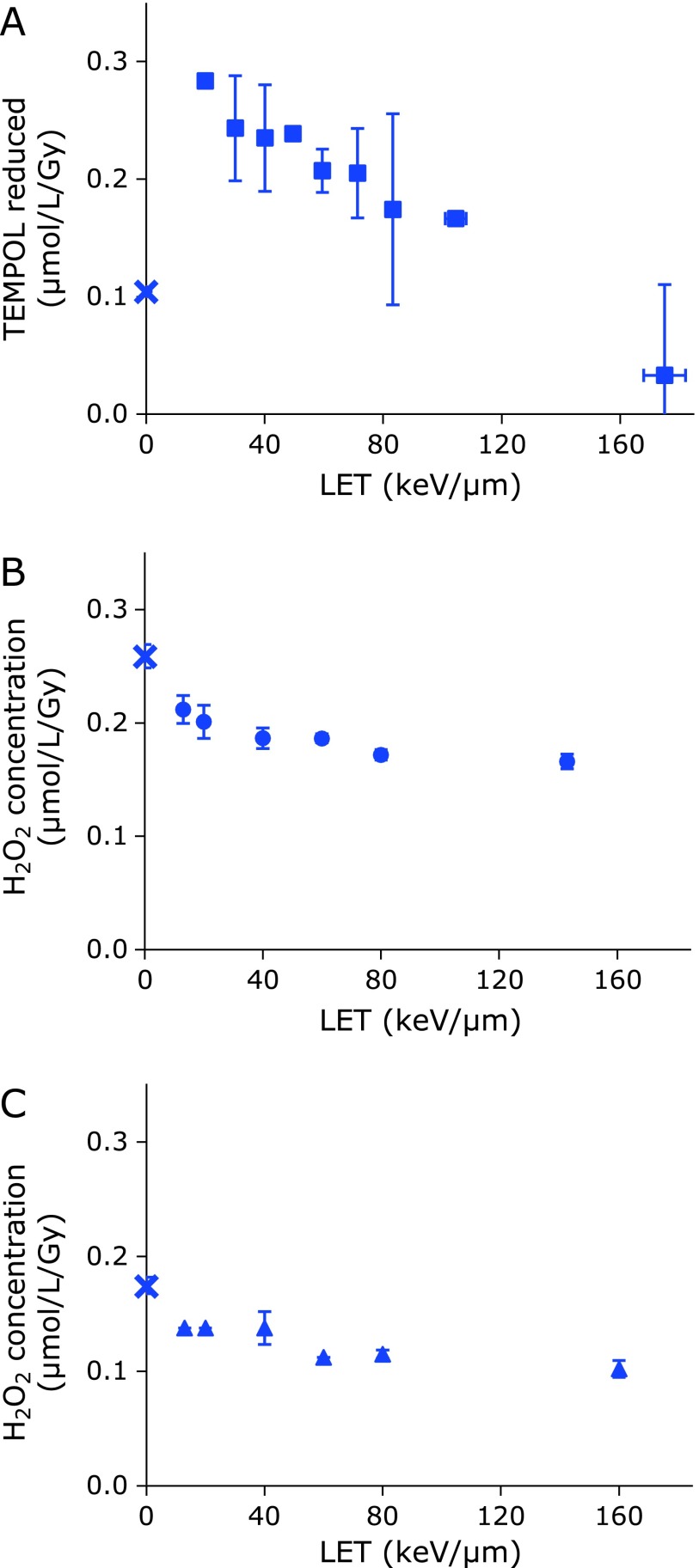
Comparison of estimation methods for radiation induced H_2_O_2_ generation. (A) Results based on direct TEMPOL reduction during irradiation in the sample solution. (B) Results based on Fenton reaction ability of irradiated water sample. (C) Results based on a spectrophotometric method. Estimated values are plotted with LET of carbon-ion beam. Marks and error bars indicate the average ± SD of 3 experiments. X mark on the Y-axis indicates result of X-ray.

**Fig. 5:**
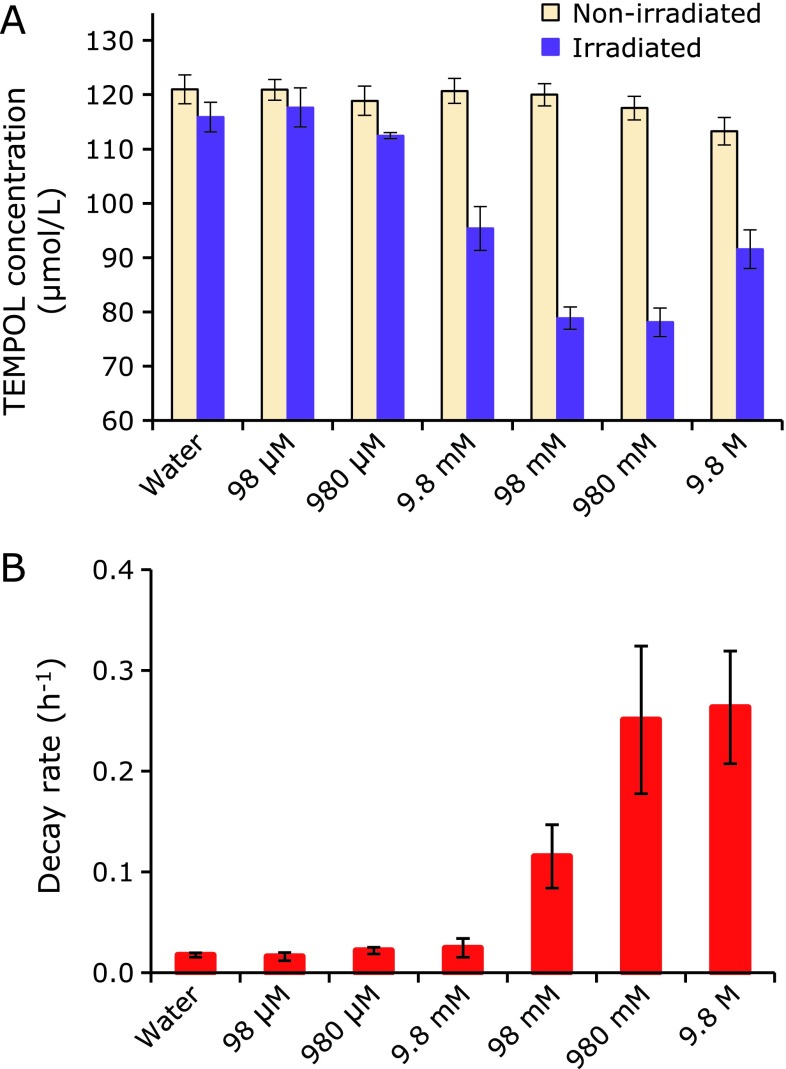
Reduction of TEMPOL induced by oxidative stimulation in several H_2_O_2_ atmospheres. (A) X-band EPR signal intensities of reaction mixtures containing 0.1 mmol/L TEMPOL and several concentrations of H_2_O_2_ were measured before and after UV irradiation at room temperature. (B) Time courses of X-band EPR signal intensities of reaction mixtures containing 0.1 mmol/L TEMPOL and several concentrations of H_2_O_2_ were measured after 1 mmol/L K_3_Fe(CN)_6_ was added at room temperature. Columns and bars indicate average and SD of 3 or 4 samples. UVB light was irradiated for 2 min at 12,000 µW/cm^2^. For reduction of TEMPOL induced by Fe^3+^, the decay rates were obtained.

Figure 4A shows a LET dependence of the amount of the GSH-independent reduction of TEMPOL, which is an H_2_O_2_ related reaction and be partly suppressed by adding catalase.^(14)^ This may be an index of H_2_O_2_ generation in the aqueous sample irradiated by ionizing radiation, leaving aside the proportionality to the H_2_O_2_ amount. The GSH-independent reduction of TEMPOL triggered by UVB irradiation or Fe^3+^ was caused only in the reaction mixture containing high concentration H_2_O_2_ (Fig. 5). Therefore, this GSH-independent reduction of TEMPOL may be indexing highly concentrated H_2_O_2_ atmosphere caused in the irradiated samples. Such a highly concentrated H_2_O_2_ atmosphere may be possible in the irradiated samples, if localized extremally dense ^•^OH generations in the irradiated aqueous samples were considered. The mechanisms of this GSH-independent reduction of TEMPOL is still in progress.

Figure 4B shows a LET dependence of the Fenton reaction ability in the irradiated water samples, which was detected by DMPO-OH generation when DMPO and Fe^2+^ were added to the irradiated water sample. Since the Fenton reaction ability was proportional to the H_2_O_2_ contents in the water sample, the amount of H_2_O_2_ generated in the irradiated water samples could be quantified using a previously prepared calibration curve. The amount of H_2_O_2_ generations decreased depending on increasing LET, when an identical dose was given. Figure 4C shows a LET dependence of the H_2_O_2_ contents in the irradiated samples measured based on a spectrophotometric method. The amount of H_2_O_2_ generations also decreased depending on increasing LET, when an identical dose was given.

The results of H_2_O_2_ generation, oxygen consumption, and the ratio of H_2_O_2_ generation/O_2_ consumption induced by X-ray and LET carbon-ion beam irradiation are summarized in Table 1. The ratio of H_2_O_2_ generation/O_2_ consumption seemed to increase with increasing LET, indicating that higher LET beam irradiation can generate H_2_O_2_ oxygen independently.

Figure 5 shows TEMPOL decay in the reaction mixture containing several different H_2_O_2_ concentrations. The EPR signal of TEMPOL was decreased in the solution containing quite high concentration (mol/L level) of H_2_O_2_, when UVB was irradiated or Fe^3+^ was added to the reaction mixture. However, in the solution containing lower H_2_O_2_ concentration (<mmol/L level), the EPR signal intensity of the TEMPOL was stable even when the UVB was irradiated or Fe^3+^ was added. Oxidative stimulation by UV irradiation or addition of Fe^3+^ could cause TEMPOL reduction in a very high concentration H_2_O_2_ environment. Therefore, the result of Fig. 4A may be indicating the dense H_2_O_2_ generation in the irradiated TEMPOL water solutions. However, the detail mechanisms of this reaction were still in progress, it is going to find out somewhere in the future.

**Fig. 6:**
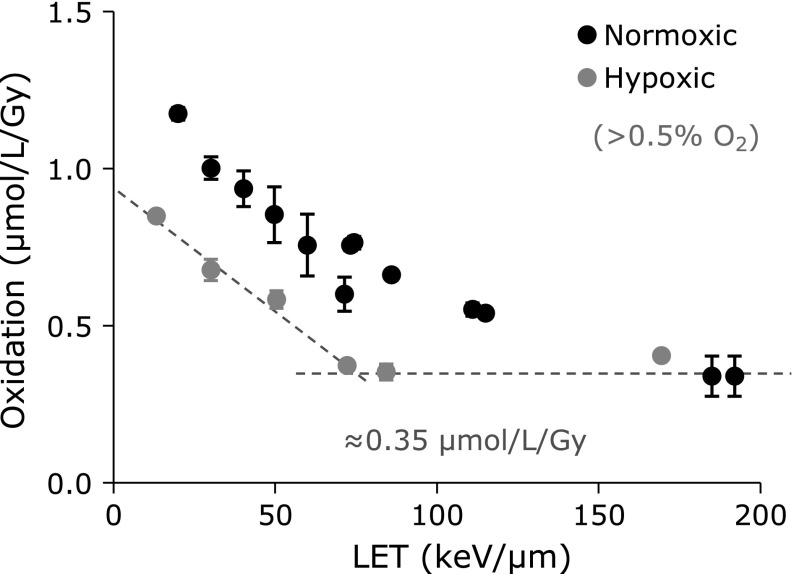
LET dependences of carbon-ion beam induced total oxidation reaction. Marks and error bars indicate average ± SD of three experiments. Black and gray marks indicate the results of normoxic and hypoxic experiments, respectively. Hypoxic concentration of the sample was monitored using an O_2_ indicator. A dotted line in the figure indicates the level of 0.35 µmol/L/Gy, which may be a contribution of ^•^OH.

Figure 6 shows the LET dependence of the amount of total oxidation reactions. For carbon-ion beam irradiation, the amount of total oxidation decreased depending on increasing LET, when an identical dose was given. Total oxidation caused by carbon-ion beam irradiation was less than that with X-ray irradiation, when the same dose was given. When the same experiment was done in a hypoxic conditions (gray circles), the amount of total oxidation decreased in the lower LET region. However, the amount of total oxidation in hypoxic conditions was not changed in the higher LET region. The horizontal dotted line in Fig. 6 indicates the level of 0.35 µmol/L/Gy, which can be considered as a contribution of ^•^OH for carbon-ion beam irradiation higher than a LET of 70 keV/µm. The other sloped dotted line may be a contribution of ^•^OH for the beam irradiation lower than LET of 70 keV/µm. Extra oxidation may probably be caused by HO_2_^•^. The values of total oxidations were summarized in Table 1.

**Fig. 7:**
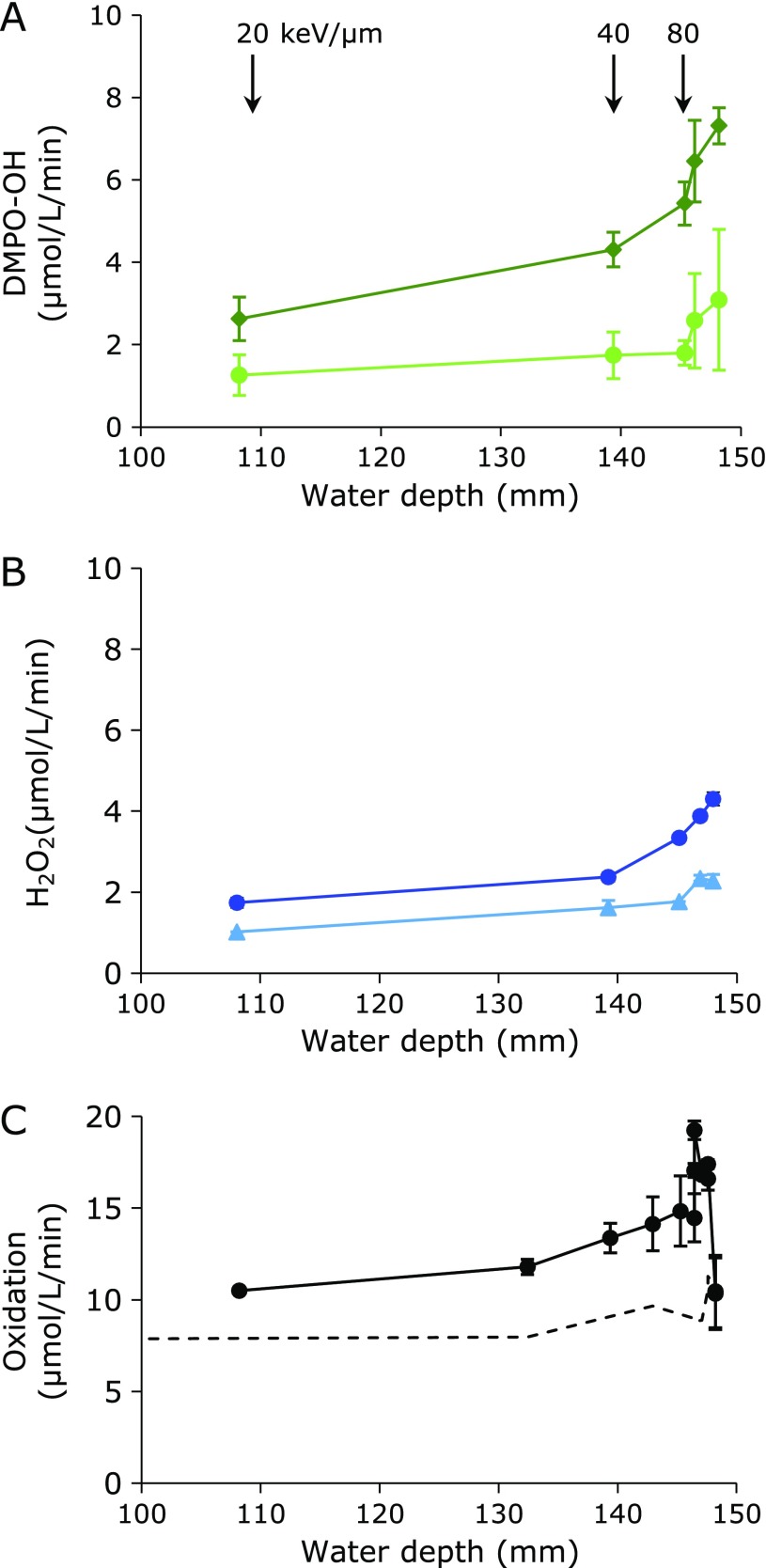
Distribution of ROS or ROS-related oxidation in a solid sample. (A) Distribution of dense (diamond) and sparse (circle) ^•^OH. (B) Distribution of H_2_O_2_. The circles and triangles were results obtained by Method 2 (modified method of EPR spin trapping) and 3 (absorption at 505 nm of the red quinoid dye), respectively. (C) Distribution of total oxidation reactions. The data shown in Fig. 1, 2, and 3 were standardized using an identical irradiation time and replotted as (A), (B), and (C). Estimated LETs at the corresponding depth are indicated at the top of (A). Dose rates were around 8.3 ± 0.99, 13.8 ± 0.56, and 23.0 ± 3.23 Gy/min at 20, 40, and 80 keV/µm, respectively. Dotted line in (C) is indicates the predicted contribution of ^•^OH.

Figure 7 shows the estimated distributions of ROS or ROS-induced oxidation reactions in one solid sample after carbon-ion mono-beam irradiation. The data shown in Fig. 2, 4, and 6 were standardized using an identical irradiation time (60 s) and replotted as Fig. 7A, B, and C, which show the distributions of ^•^OH, H_2_O_2_, and total oxidation reactions, respectively. The distributions of ^•^OH generation were estimated for the first time. The amount of the total ^•^OH at the position close to the Bragg peak was much larger compared with the sparse ^•^OH, H_2_O_2_, and total oxidation reactions. This dense ^•^OH generation may reflect the dose of radiation at that point. The distribution of ROS increased gradually with increasing water depth, and had a peak around 146–148 mm, which may be close to the position of the Bragg peak. The distribution of ROS must depend on both the LET dependence of ROS generation and the distribution of the dose. The dose rates were around 8.3 ± 0.99, 13.8 ± 0.56, and 23.0 ± 3.23 Gy/min at 20, 40, and 80 keV/µm, respectively. The dotted line in Fig. 7C is the estimated contribution of ^•^OH, which was a calculated based on the oxidation values observed under hypoxic atmosphere, i.e., gray marks in Fig. 6. The deduction of ^•^OH contribution from total oxidation reactions would probably be oxidation due to HO_2_^•^.

Applying the result of ^•^OH generation to a known ionization structure with low LET radiation, we can have an image of the molecular distribution of ^•^OH generation. For sparse ^•^OH generation, the distance between ^•^OH and another ^•^OH was calculated as 4.3–6.6 nm based on the estimated density. More than 14 water molecules can exist in such a 4.3 nm gap. In this model, the sparse ^•^OH may be eliminated before reaching any organic molecules because of the distance. However, hydrogen radicals (^•^H), a counterpart of ^•^OH, can react with oxygen and give HO_2_^•^, which may be more important for low LET radiation. In dense ^•^OH clusters, the distance between ^•^OH and the next ^•^OH can be less than 1 nm; therefore, ^•^OH may be able to react with another and give H_2_O_2_. Again, a reaction of H_2_O_2_ with another ^•^OH gives HO_2_^•^. Therefore, low LET radiation may mainly generate a sparse O_2_^•−^/HO_2_^•^ atmosphere, which is dependent on resolved-oxygen in the reaction mixture.

Track structures reported for heavy-ion beams are composed of a core and a penumbra.^(26)^ Sparse ^•^OH generation such as low LET radiation can be expected in the penumbra region, and much denser ^•^OH generation can be expected in the core region. It can be expected from the result in Fig. 2 that low LET radiation such as ^•^OH generation in the penumbra may be reduced according to the increase in LET. As a result, extremely dense ^•^OH in the track core can occur especially in the high LET part, as shown in Fig. 7A. When biological molecules are in the track of the carbon-ion beam, attack by dense ^•^OH may be unavoidable. The reaction of such extremely dense ^•^OH may be hardly distinguishable from the direct action of radiation. In addition, such dense and abundant ^•^OH can be settled in an H_2_O_2_ atmosphere, which can be made independently of the presence of oxygen.

^•^OH generation was not uniform and was localized at a molecular level. There were two forms of ^•^OH generation, i.e., sparse ^•^OH and dense ^•^OH. The ratio of sparse and dense ^•^OH generation was LET dependent. In dense ^•^OH conditions, ^•^OH molecules can react with each other and make H_2_O_2_ and HO_2_^•^ in an O_2_-independent manner. It can be expected that differences in ROS generation and distribution could make a difference in the quality of radiation therapy.

## Conclusion

Radiation-induced ROS generation was quantified. Two different forms of ^•^OH generation, dense and sparse generation, as described as previous papers,^(12,13)^ were estimated separately. Total ^•^OH generation was expected to be identical at the same dose even for X-ray or carbon-ion beam irradiation. Sparse ^•^OH generation decreased with increasing LET. Carbon-ion beam irradiation produced denser H_2_O_2_ compared with X-ray irradiation, although the ratio of H_2_O_2_ generation per oxygen consumed increased with LET. Total oxidation reactions decreased with increasing LET. ROS generation was not uniform and was LET dependent at the molecular level.

## Figures and Tables

**Table 1:** Breakdown of ROS generation by X-ray and carbon-ion beam irradiation

	X-ray	Carbon-ion beam				
		20 keV/µm	40 keV/µm	60 keV/µm	80 keV/µm	>100 keV/µm^a^
Total ^•^OH generation^b^ (µmol/L/Gy)	0.35 ± 0.01	0.33 ± 0.02	0.31 ± 0.01	0.34 ± 0.01	0.30 ± 0.00	0.28 ± 0.01
Sparse ^•^OH generation^c^ (µmol/L/Gy)	0.17 ± 0.02	0.15 ± 0.03	0.11 ± 0.02	0.13 ± 0.05	0.12 ± 0.04	0.09 ± 0.02
Density of sparse ^•^OH^d^ (µm^−1^)	149 ± 15	142 ± 6	136 ± 18	150 ± 37	155 ± 31	153 ± 28
Concentration of sparse ^•^OH^e^ (mM)	5.6 ± 1.7	4.7 ± 0.6	4.3 ± 1.7	6.1 ± 4.2	6.6 ± 3.7	6.2 ± 3.3
H_2_O_2_ generation^f^ (µmol/L/Gy)	0.26 ± 0.010	0.20 ± 0.015	0.19 ± 0.009	0.19 ± 0.004	0.17 ± 0.005	0.17 ± 0.006
O_2_ consumption^g^ (µmol/L/Gy)	0.41 ± 0.042	0.39 ± 0.145	0.28 ± 0.019^h^	0.23 ± 0.031	0.15 ± 0.059	0.05 ± 0.125
H_2_O_2_/O_2_ consumption	0.63	0.51	0.68	0.83	1.13	3.40
Total oxidation (µmol/L/Gy)	2.74 ± 0.217	1.17 ± 0.020	0.94 ± 0.057	0.76 ± 0.098	0.66 ± 0.004	0.34 ± 0.064

^a^LET was not actually 100 keV/µm, but was more than 100 keV/µm. ^b^Total ^•^OH denotes the calculated Y-value at the DMPO density of 1,000 µm^−1^ on the least square line for 3rd phase. ^c^Sparse ^•^OH generation and the ^d^density of sparse ^•^OH was calculated from the intersection point of least square lines obtained for first and second phases of the plot of DMPO-OH concentration vs DMPO density. ^e^The density of sparse ^•^OH was replaced with the corresponding concentration. ^f^H_2_O_2_ generation values were results of Method 2. ^g^The values of O_2_ consumption were referenced from a previous paper.^(14)^ ^h^Result at LET = 50 keV/µm.
